# Widely targeted metabolic profiling reveals drought resistance mechanisms in alfalfa leaves

**DOI:** 10.1186/s12870-025-07163-9

**Published:** 2025-09-22

**Authors:** Wenjuan Wang, Wenjuan Kang, Shangli Shi, Linbo Liu

**Affiliations:** Key Laboratory of Grassland Ecosystem, Gansu Agricultural University, Ministry of Education, Lanzhou, China

**Keywords:** Alfalfa, Drought stress, Phenotypic, Metabolomics, Drought-resistant mechanisms

## Abstract

Drought is considered a primary factor constraining alfalfa (*Medicago sativa* L.) yield and acreage. To understand the internal drought resistance mechanisms of alfalfa is essential for breeding drought-resistant alfalfa varieties. Here, we compared the phenotypic characteristics, metabolic pathways and metabolites of drought-resistant (Longzhong, LZ) and drought-sensitive (Gannong No. 3, G3) varieties under drought stress. Phenotypic analysis revealed that drought stress reduced plant height, single plant fresh weight, single plant dry weight and leaf RWC in alfalfa, with a greater effect observed in G3. The root length of LZ increased under drought stress, whereas there was no significant change in the root length of G3. Widely targeted metabolomics revealed that LZ could maintain higher glycolysis/gluconeogenesis and tricarboxylic acid cycle under drought stress, which provided more ATP and substrates for amino acids biosynthesis, arginine and proline metabolism and phenylpropanoid metabolism. This allowed LZ to accumulate more amino acids, spermidine, spermine, 4-aminobutyric acid, naringenin, isoliquiritigenin, glycitein, glycitin, calycosin, ferulate, scopoline, scopoletin, sinapyl alcohol and coniferin, which favor the enhancement of drought resistance in alfalfa. Moreover, widely targeted metabolomics showed salicylic acid and trans-zeatin were key hormones involved in drought resistance in alfalfa. The results of this study provide useful insights into the enhancement of drought resistance in alfalfa through metabolic regulatory mechanisms, which provides a theoretical basis for the breeding drought-resistant alfalfa varieties.

**Supplementary Information:**

The online version contains supplementary material available at 10.1186/s12870-025-07163-9.

## Introduction

Drought events are becoming more severe and frequent as a result of global climate change due to the greenhouse effect [1]. It is estimated that more than 50% of the reduction in crop yields caused by abiotic stress is due to drought [2]. Thus, drought has become the most important abiotic stress limiting crop growth and yield globally [3]. Drought stress has significant negative effects on morphological, biochemical, physiological and molecular processes in plants, including by decreasing photosynthesis, increasing oxidative stress, damaging plasma membranes and cells, perturbing cellular metabolism, damaging plant organs, and even causing plant death [4–6]. To alleviate the harmful effects of drought, plants have evolved multiple mechanisms to combat drought, including via maintenance of the structure and properties of the biological membrane [7], enhancement of antioxidant defences [8], and increased accumulation of osmoregulatory substances in the cell [9].

In addition, plants can adjust and regulate metabolic networks to induce the production of a series of specialized metabolites to defend against stress [10]. Metabolomics is an emerging technology involving qualitative or quantitative analysis of plant metabolite contents. Plants metabolomics make essential contributions to the study of stress biology by recognizing various compounds like by-products of stress metabolism, stress signaling molecules and other molecules that are part of the plant adaptation process [11]. For example, metabolomic results have revealed that pentitol, phytol, xylonic acid, D-xylopyranose, stearic acid and D-ribose are important metabolites for drought resistance in peanut. Among them, agmatine, cadaverine, and polyphenols such as syringic acid and vanillic acid are more favourable for enhancing the drought resistance of peanut [12]. Under drought stress, galactose metabolism, the tricarboxylic acid (TCA) cycle, aminoacyl-tRNA biosynthesis, and nicotinate and nicotinamide metabolism were found to be more active in the drought-resistant oilseed rape variety than in the drought-sensitive oilseed rape variety. The accumulation of galactose, tagatose, glycerolone and fumaric acid favours drought stress resistance in oilseed rape [13]. Metabolomic results revealed more pronounced increases in amino acid, flavonoid, organic acid and soluble sugar contents in drought-resistant asparagus varieties than in drought-sensitive asparagus varieties [14]. These metabolomics studies revealed key metabolic pathways and metabolites that increase drought stress resistance in plants.

Alfalfa (*Medicago sativa* L.) is a perennial leguminous forage grass that is widely grown around the world for its high yield, quality and palatability. Drought is recognized as a major factor limiting alfalfa yield and acreage [15]. Therefore, exploring the protective mechanisms of alfalfa against drought stress is crucial for breeding alfalfa for drought resistance. Numerous studies on the mechanism of drought resistance in alfalfa have focused mainly on changes at the phenotypic and physiological levels. A previous nontargeted metabolomic study on the root systems of three types of alfalfa with different drought resistance revealed that gibberellin A4 (GA4), indole-3-acetic acid (IAA), abscisic acid (ABA), salicylic acid (SA), 6-gingerol, sucrose, L-tyrosine, L-phenylalanine, succinic acid, trans-cinnamic acid and nicotinic acid were necessary for the resistance of the three types of alfalfa to drought stress [16]. However, few studies have revealed the metabolic pathways and metabolites associated with drought resistance in alfalfa leaves through widely targeted metabolomics. Widely targeted metabolic profiling is one of the most advanced tools for qualitatively and quantitatively characterizing changes in all metabolites in plants due to stress or perturbation [17].

In this study, we compared the differences in phenotype and metabolic changes in the leaves of alfalfa varieties with different drought resistance under drought stress via a widely targeted metabolic profiling technique based on ultrahigh-performance liquid chromatography-mass spectrometry (UHPLC-MS/MS) to clarify the metabolic network involved in the response of alfalfa to drought stress. It can understand and supplement the metabolic mechanism of drought resistance in alfalfa, thus providing new thoughts for the study of improving drought resistance in alfalfa through metabolic regulation.

## Materials and methods

### Plant materials and experimental conditions

Two alfalfa varieties with different drought resistance were used in this study: *Medicago sativa* L. cv. Longzhong (LZ), which is a drought-resistant variety, and *Medicago sativa* L. cv. Gannong No. 3 (G3), which is a drought-sensitive variety. Seeds of LZ and G3 were provided by the Grassland Ecosystems Key Laboratory, Ministry of Education of China. In accordance with our previously described method [18], the seeds were sown in 9 cm × 11 cm plastic cups containing 450 g of sand and then put into a 25 cm × 15 cm × 10 cm nutrient bowl containing 300 mL of distilled water to maintain sufficient moisture in the sand to provide the proper humidity for seed germination. Each nutrient bowl contained six plastic cups. Twelve alfalfa seedlings were planted in each plastic cup. The nutrient bowls were then placed in growth chambers set up under the following conditions: relative humidity of 45%, photosynthetic flux density of 450 µmol m^−2^ s^−1^, day/night temperature of 25/18°C, and light/dark photoperiods of 16/8 h. After all the seeds had germinated, 500 mL of nutrient solution was added to the nutrient bowl weekly. The seedlings were divided into two groups for each alfalfa variety after 42 days of seedling growth. One group was the drought-treated group, in which alfalfa seedlings grown for 42 days were watered every other day with 300 ml of PEG-6000 nutrient solution with an osmotic pressure of −1.2 MPa in each nutrient bowl. The other group was the control group, which was well watered with Hoagland solution without PEG-6000. Therefore, four treatments were established in this experiment: LZ-Control, LZ-Drought, G3-Control and G3-Drought. On day 8 of the drought treatment, healthy leaves were collected from all the treatment groups described above and then immediately frozen in liquid nitrogen and stored at −80 °C for widely targeted metabolic analysis.

### Measurement of phenotypic characteristics

The plant height and root length (main root length, the length of the longest root in the vertical downward direction) of alfalfa were measured with a ruler (1/100), and each treatment was repeated five times. The single plant fresh weight and single plant dry weight were determined using an electronic balance (1/10000), and each treatment was repeated five times. The leaf relative water content (RWC) were determined according to our previously described methods [19], and each treatment was repeated three times.

### Metabolite extraction and quantification

Metabolomic analysis was performed at Novogene Co., Ltd. (Beijing, China). The detailed steps of UHPLC‒MS/MS analysis as well as metabolite identification and quantification were as described in previous studies [17].

### Statistical analysis

Phenotypic characteristics data and the relative contents of metabolites were analysed using GraphPad Prism 8.0.2 software, and significant differences were determined using Tukey’s test and two-way ANOVA. *p* < 0.05 represented statistical significance. PCA and partial least squares discriminant analysis (PLS-DA) analyses of the metabolomic data were performed using metaX software. Volcano plots were generated with ggplot2 in R. Redundancy analysis (RDA) analysis of metabolites was performed using canoco5 software.

## Results

### Phenotypic characteristics

**Fig. 1:**
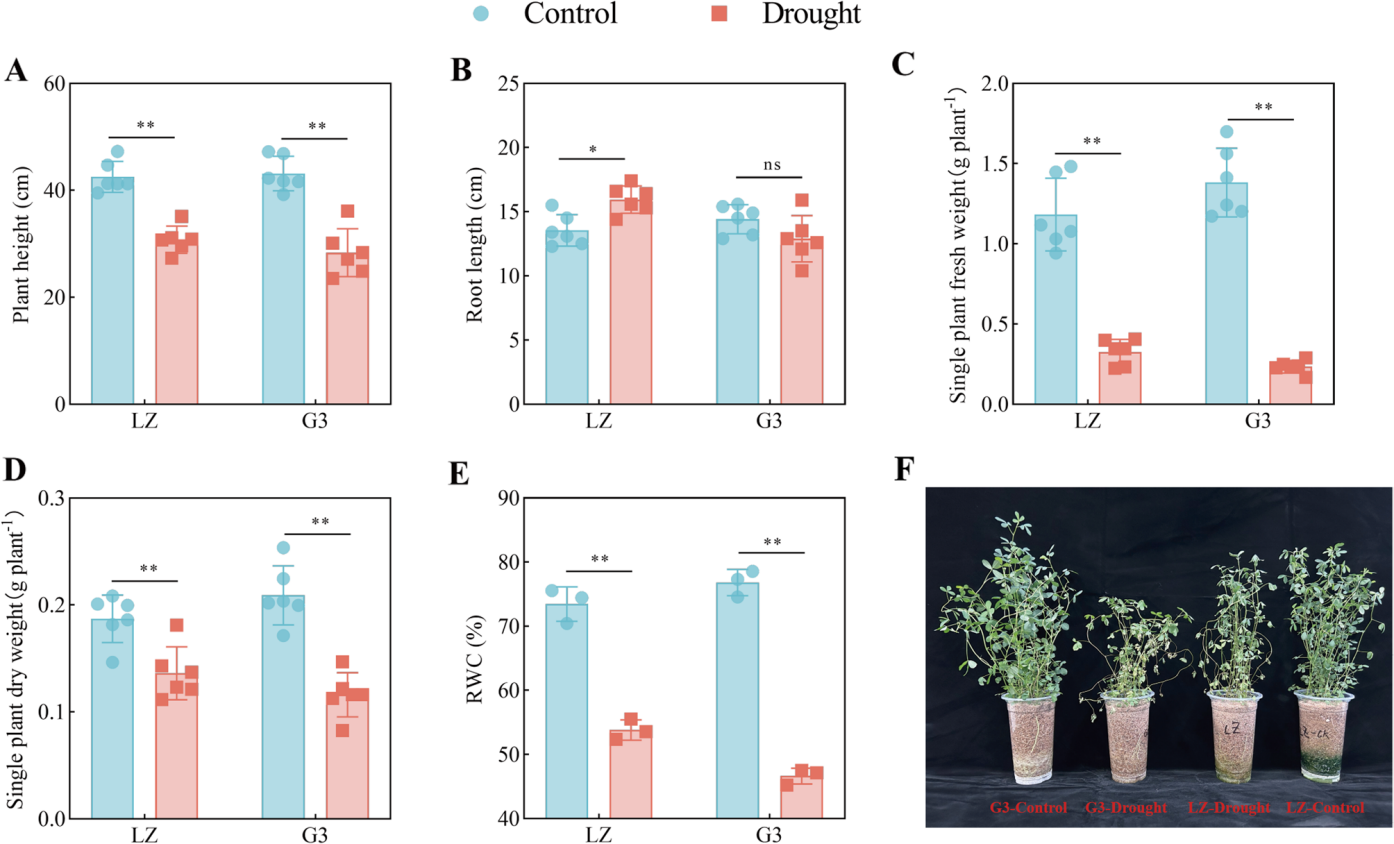
Effects of drought stress on the phenotypic indices of alfalfa varieties with different drought resistance. **A** Plant height. **B** Root length. **C** Single-plant fresh weight. **D** Single-plant dry weight. **E** RWC. **F** Growth conditions of the plants in all the treatments in the experiment. From left to right: G3-Control, G3-Drought, LZ-Drought, LZ-Control. “ns”: no significant difference between the two treatments (*p* > 0.05); “*”: significant difference between the two treatments (*p* < 0.05); “**”: extremely significant difference between the two treatments (*p* < 0.01). The same applies below

As shown in Fig. 1, compared with the control, drought significantly reduced plant height, single-plant fresh weight, single-plant dry weight and leaf RWC, which decreased by 27.63%, 72.47%, 27.20% and 26.75%, respectively, in LZ and by 34.30%, 83.07%, 44.4% and 39.29%, respectively, in G3. Under drought stress, the root length of LZ significantly increased by 17.71%, but there was no significant increase in the root length of G3.

### Search for differentially abundant metabolites (DAMs)

**Fig. 2:**
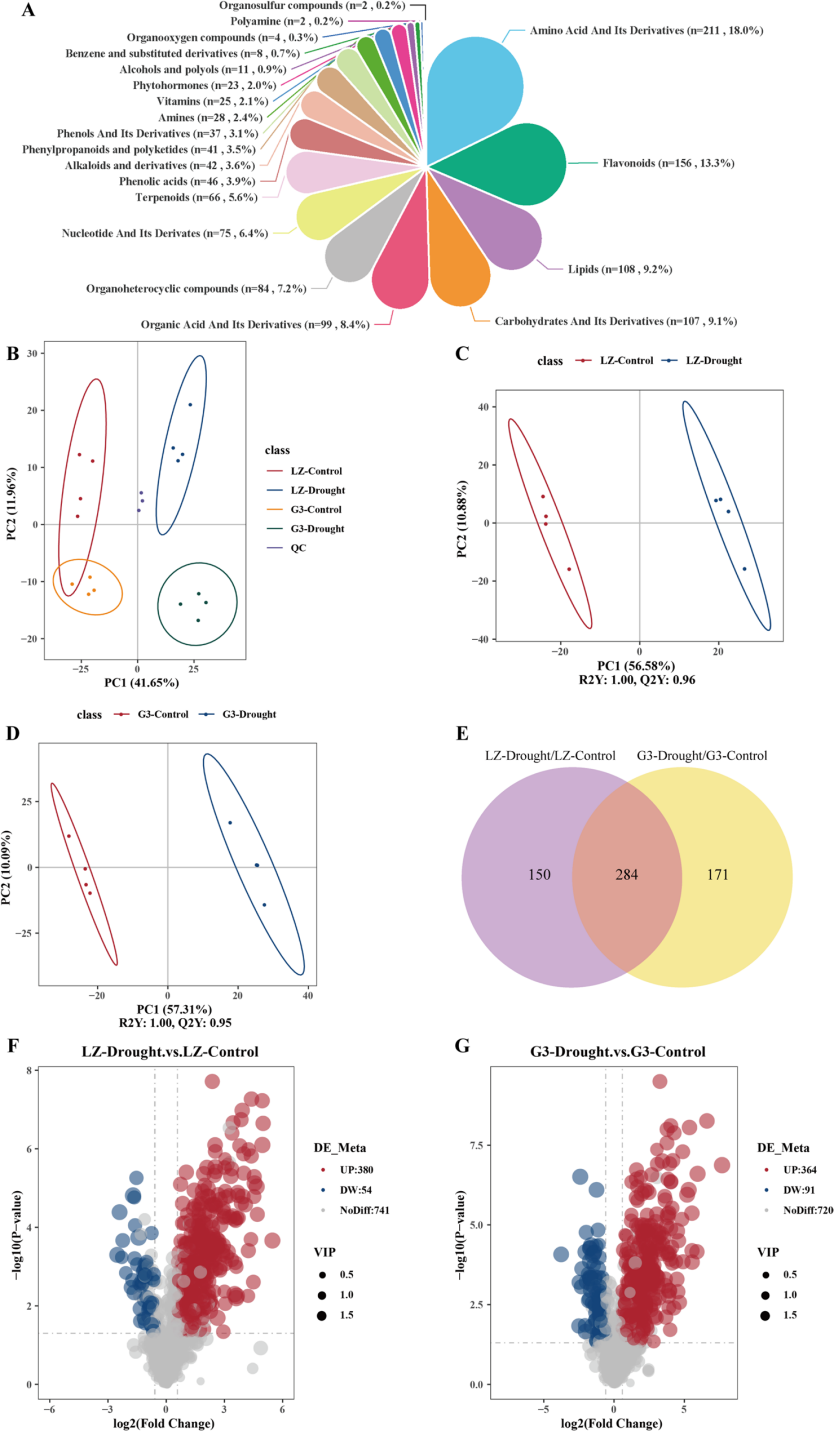
**A** Metabolite classification pie chart. **B** PCA of total samples. **C** PLS-DA of LZ-Control and LZ-Drought. **D** PLS-DA of G3-Control and G3-Drought. **E** Venn diagram analysis of the DAMs. **F** DAM volcano plots for LZ-Control vs. LZ-Drought. **G** DAM volcano plots for G3-Control vs. G3-Drought

Widely targeted metabolomics was performed on two alfalfa varieties with different drought resistance under control and drought conditions, and a total of 1,175 metabolites were identified (Supplementary Table 1). These metabolites were mainly divided into amino acid and its derivatives (17.96%), flavonoids (13.28%), lipids (9.19%), carbohydrates and its derivatives (9.11%), organic acid and its derivatives (8.43%), organoheterocyclic compounds (7.15%), nucleotide and its derivates (6.38%), terpenoids (5.62%), phenolic acids (3.91%), alkaloids and derivatives (3.57%), phenylpropanoids and polyketides (3.49%), phenols and its derivatives (3.15%), amines (2.38%), and vitamins (2.13%) (Fig. 2A). Principal component analysis (PCA) revealed a distinct separation between the control and drought treatments in both varieties. The first two PCs explained 53.61% of the total variation. PC1 accounted for 41.65% of the total data variance, whereas PC2 explained 11.96% of the data variance for the entire dataset (Fig. 2B). PLS-DA was performed for the control and drought treatments for each variety. The results indicated that R2 was 1.0 and Q2 was 0.96 for LZ, and R2 was 1.0 and Q2 was 0.95 for G3, which reflected the accuracy and reliability of the model (Fig. 2C, D). A VIP > 1.0, FC > 1.5 or FC < 0.667 and P value < 0.05 were used as set thresholds to screen for DAMs. In the LZ-Drought/LZ-Control comparison group, there were 434 DAMs, of which 380 were upregulated and 54 were downregulated, and in the G3-Drought/G3-Control comparison group, there were 455 DAMs, of which 364 were upregulated and 91 were downregulated (Fig. 2E, F, G).

### Bubble diagram of enriched pathways for dams

**Fig. 3:**
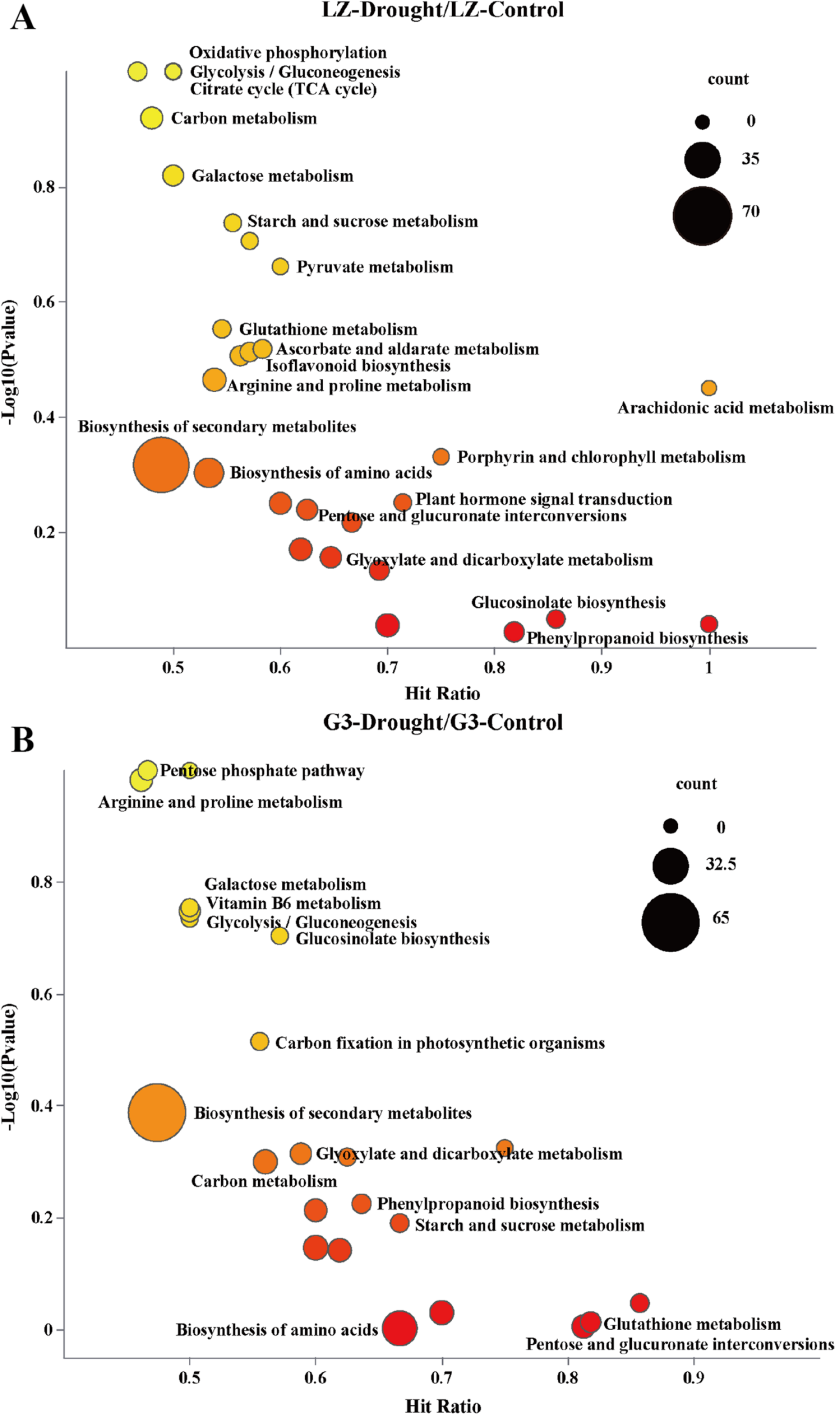
**A** KEGG enrichment bubble plots for LZ-Control/LZ-Drought. **B** KEGG enrichment bubble plots for G3-Control/G3-Drought

KEGG enrichment can identify the most important biochemical metabolic pathways associated with DAMs. The major pathways enriched in the LZ-drought/LZ-control comparison group were biosynthesis of secondary metabolites, biosynthesis of amino acids, pentose and glucuronate interconversions, plant hormone signal transduction, glucosinolate biosynthesis, arginine and Pro metabolism, isoflavone biosynthesis, glycolysis/gluconeogenesis, the citrate cycle (also known as the TCA cycle) and oxidative phosphorylation (Fig. 3A). The major pathways enriched in the G3-drought/G3-control comparison group were biosynthesis of secondary metabolites, biosynthesis of amino acids, pentose and glucuronate interconversions, glutathione metabolism, starch and sucrose metabolism, carbon metabolism, glyoxylate and dicarboxylate metabolism, porphyrin and chlorophyll metabolism, glucosinolate biosynthesis, glycolysis/gluconeogenesis, vitamin B6 metabolism, galactose metabolism, arginine and Pro metabolism, the pentose phosphate pathway (PPP), and phenylpropanoid biosynthesis (Fig. 3B). In summary, we grouped the above major drought-related metabolic pathways into sugar metabolism (glycolysis/gluconeogenesis, PPP, TCA cycle), amino acid biosynthesis, arginine and Pro metabolism, phenylpropanoid biosynthesis, and plant hormone signal transduction pathways.

#### Glycolysis/gluconeogenesis and the PPP

**Fig. 4:**
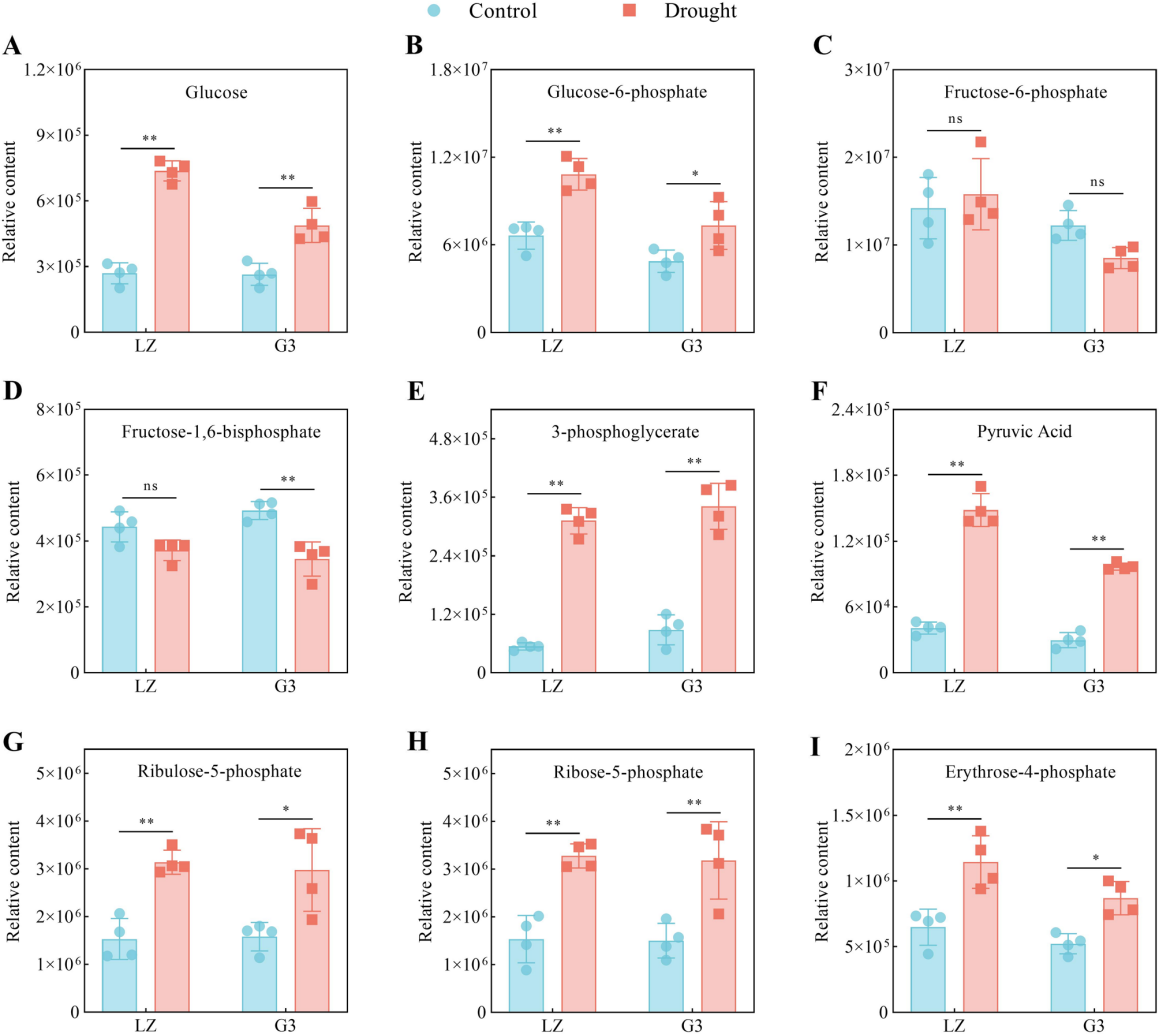
Relative contents of glycolysis/gluconeogenesis- and PPP-related metabolites. **A** Relative glucose content. **B** Relative glucose-6-phosphate content. **C** Relative fructose-6-phosphate content. **D** Relative fructose-1,6-bisphosphate content. **E** Relative 3-phosphoglycerate content. **F** Relative pyruvic acid content. **G** Relative ribulose-5-phosphate content. **H** Relative ribose-5-phosphate content. **I** Relative erythrose-4-phosphate content

As shown in Fig. 4, drought stress increased the relative contents of glucose, glucose-6-phosphate, 3-phosphoglycerate, pyruvic acid, ribulose-5-phosphate, ribose-5-phosphate and erythrose-4-phosphate in the leaves of the two alfalfa varieties, with greater increases observed in LZ than in G3. Drought stress had no significant effect on the relative fructose-6-phosphate content of the leaves of either alfalfa variety. The relative content of fructose-1,6-bisphosphate in LZ alfalfa leaves did not change significantly under drought stress, whereas that in G3 decreased significantly. These results suggested that LZ could better maintain glycolysis/gluconeogenesis and PPP under drought stress than could G3.

#### TCA cycle

**Fig. 5:**
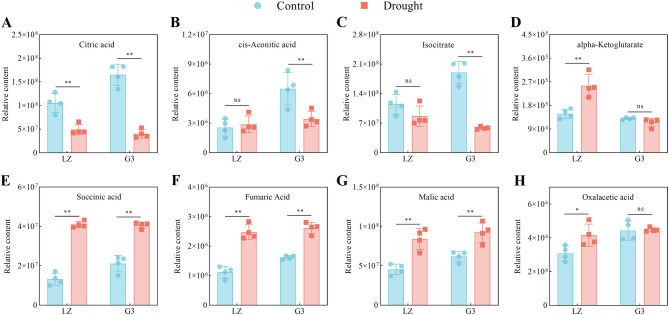
Relative levels of TCA cycle-related metabolites. **A** Relative citric acid content. **B** Relative cis-aconitic acid content. **C** Relative isocitrate content. **D** Relative alpha-ketoglutarate content. **E** Relative succinic acid content. **F** Relative fumaric acid content. **G** Relative malic acid content. **H** Relative oxalic acid content

As shown in Fig. 5, under drought stress, the alpha-ketoglutarate, succinic acid, fumaric acid, malic acid and oxalacetic acid contents of LZ alfalfa leaves significantly increased by 72.17%, 211.66%, 121.22%, 85.36% and 35.30%, respectively, and the citric acid content significantly decreased by 53.36%, whereas the contents of cis-aconitic acid and isocitrate did not change significantly. The succinic acid, fumaric acid and malic acid contents of G3 alfalfa leaves increased significantly by 92.95%, 61.61% and 49.41%, respectively, and the citric acid, cis-aconitic acid and isocitrate contents decreased significantly by 75.67%, 47.27% and 69.20%, respectively, while the alpha-ketoglutarate and oxalacetic acid contents did not change significantly. The above results indicated that, compared with G3, LZ alfalfa could maintain a better TCA cycle under drought stress.

#### Amino acid biosynthesis

**Fig. 6:**
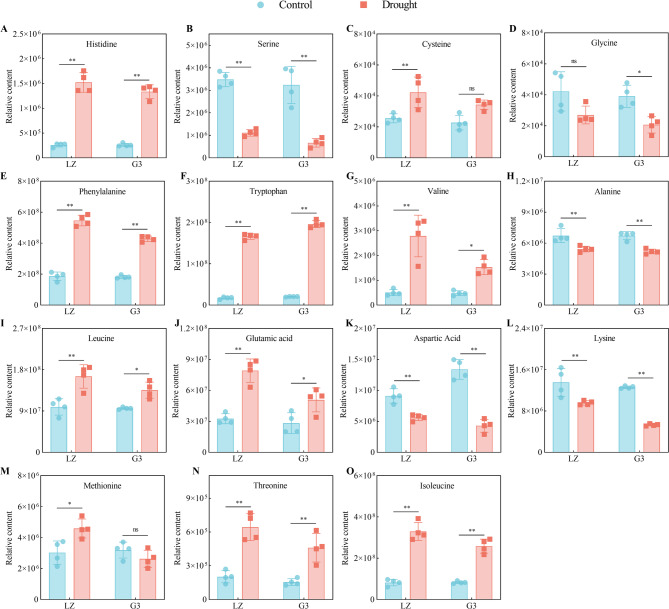
Relative contents of metabolites associated with amino acid biosynthesis. **A** Relative histidine content. **B** Relative serine content. **C** Relative cysteine content. **D** Relative glycine content. **E** Relative phenylalanine content. **F** Relative tryptophan content. **G** Relative valine content. **H** Relative alanine content. **I** Relative leucine content. **J** Relative glutamic acid content. **K** Relative aspartic acid content. **L** Relative lysine content. **M** Relative methionine content. **N** Relative threonine content. **O** Relative isoleucine content

Under drought stress, the relative contents of histidine, phenylalanine, tryptophan, valine, leucine, glutamic acid, threonine, and isoleucine in the leaves of the two alfalfa varieties increased significantly, and the increase in the contents of these amino acids in LZ was greater than that in G3; the relative contents of serine, alanine, aspartic acid and lysine in the leaves of the two alfalfa varieties decreased significantly, and the decrease in the contents of these amino acids in G3 was greater than that in LZ. Under drought stress, in LZ alfalfa leaves, the relative contents of cysteine and methionine increased significantly, while the relative content of glycine did not change significantly; however, in G3, the relative contents of cysteine and methionine did not change significantly, while the relative content of glycine decreased significantly. Overall, LZ accumulated more amino acids than did G3 under drought stress (Fig. 6A-O).

#### Arginine and pro metabolism

**Fig. 7:**
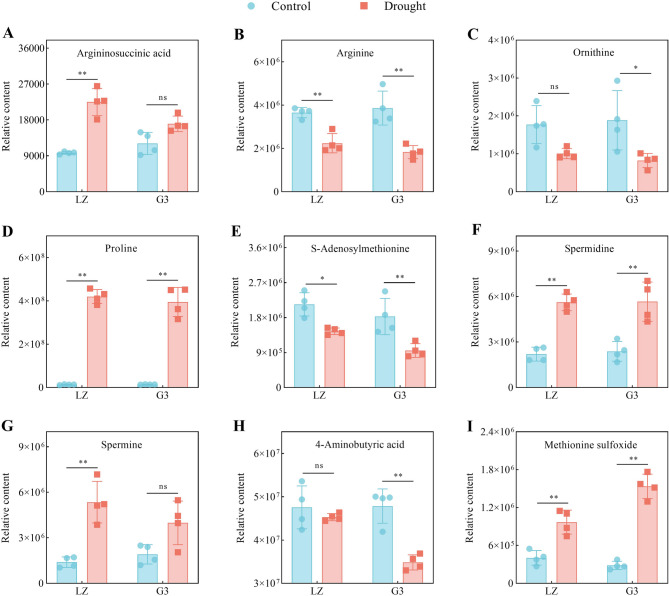
Relative contents of arginine and proline metabolism-related metabolites. **A** Relative argininosuccinic acid content. **B** Relative arginine content. **C** Relative ornithine content. **D** Relative proline content. **E** Relative S-adenosylmethionine content. **F** Relative spermidine content. **G** Relative spermine content. **H** Relative 4-aminobutyric acid content. **I** Relative methionine sulfoxide content

As shown in Fig. 7, under drought stress, the relative contents of argininosuccinic acid, Pro, spermidine (Spd) and spermine (Spm) in LZ alfalfa leaves significantly increased; the relative contents of Pro and Spd in G3 alfalfa leaves also significantly increased, but the increase was less than that in LZ; and there was no significant change in the relative contents of argininosuccinic acid and Spm in G3 alfalfa leaves. Drought stress resulted in significant decreases in the relative contents of arginine and S-adenosylmethionine in the leaves of the two alfalfa varieties, with the decreases being greater in G3 than in LZ. The relative contents of ornithine and 4-aminobutyric acid (GABA) significantly decreased in G3 under drought stress, whereas there was no significant change in the levels of these metabolites in LZ. Drought stress induced a significant increase in methionine sulfoxide in the leaves of the two alfalfa varieties, with the increase in the methionine sulfoxide level in G3 being greater than that in LZ. The above results indicated that LZ accumulated more Pro, Spd, Spm and GABA and less methionine sulfoxide under drought stress than did G3.

#### Phenylpropanoid biosynthesis

**Fig. 8:**
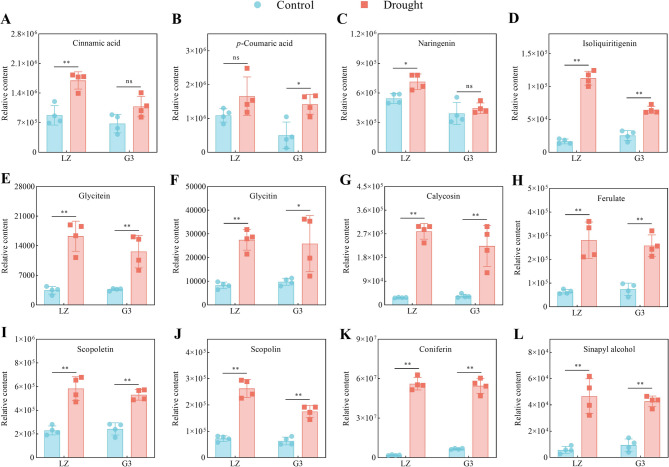
Relative contents of phenylpropanoid biosynthesis-related metabolites. **A** Relative cinnamic acid content. **B** Relative p-coumaric acid content. **C** Relative naringenin content. **D** Relative isoliquiritigenin content. **E** Relative glycitein content. **F** Relative glycitin content. **G** Relative calycosin content. **H** Relative ferulate content. **I** Relative scopoletin content. **J** Relative scopoline content. **K** Relative coniferin content. **L** Relative sinapyl alcohol content

As shown in Fig. 8, drought stress significantly increased the relative contents of isoliquiritigenin, glycitein, glycitin, calycosin, ferulate, scopoletin, scopoline, coniferin and sinapyl alcohol in the leaves of the two alfalfa varieties, with the increase in the levels of these metabolites in LZ being greater than that in G3. Under drought stress, the relative contents of cinnamic acid and naringenin in LZ alfalfa leaves increased significantly, and the relative content of *p*-coumaric acid did not change significantly; however, the relative contents of cinnamic acid and naringenin in G3 alfalfa leaves did not change significantly, and the relative content of *p*-coumaric acid increased significantly. These results suggested that LZ accumulated more phenylpropanoids under drought stress than did G3.

#### Plant hormones

**Fig. 9:**
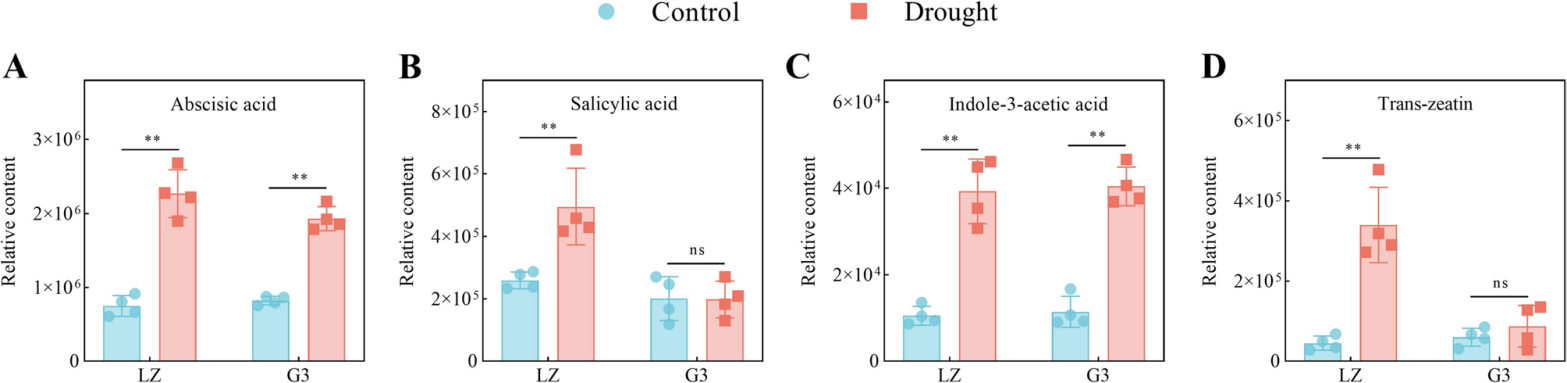
Relative contents of plant hormone signal transduction-related metabolites. **A** Relative abscisic acid content. **B** Relative salicylic acid content. **C** Relative indole-3-acetic acid content. **D** Relative *trans*-zeatin content

As shown in Fig. 9, drought stress caused a significant increase in the relative contents of ABA and IAA in the leaves of the two alfalfa varieties, with the increase in the levels of these metabolites in LZ being greater than that in G3. The relative contents of SA and trans-zeatin (TZ) in LZ alfalfa leaves significantly increased under drought stress, whereas those in G3 did not significantly change. These results indicated that LZ accumulated more ABA, SA, IAA and TZ under drought stress than did G3, especially ABA, SA and TZ.

#### RDA of dams

**Fig. 10:**
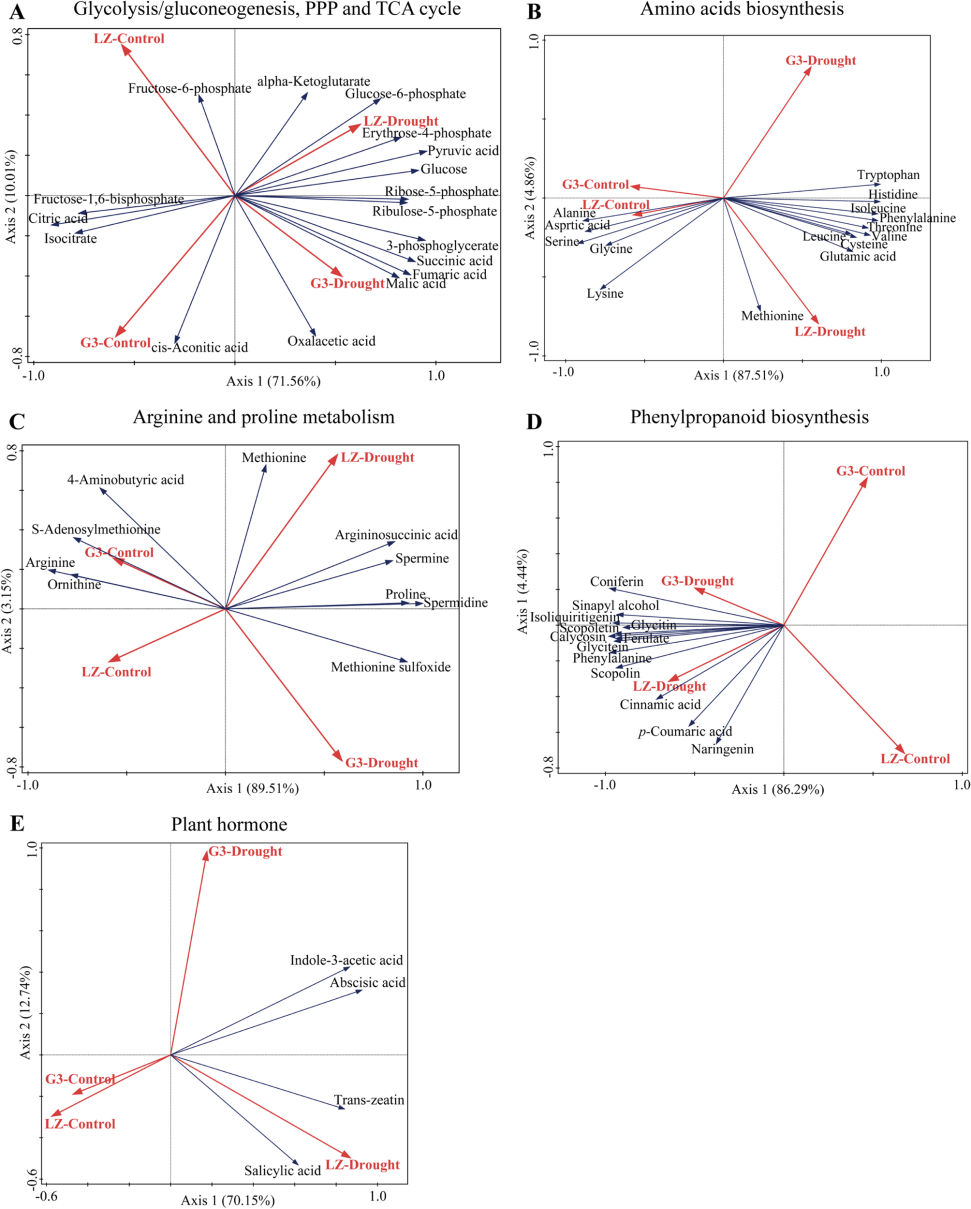
RDA of DAMs. **A** Glycolysis/gluconeogenesis, the PPP and the TCA cycle. **B** Amino acid biosynthesis. **C** Arginine and proline metabolism. **D** Phenylpropanoid biosynthesis. **E** Plant hormones

As shown in Fig. 10, RDA of the DAMs in the above metabolic pathways revealed that glucose-6-phosphate, pyruvic acid and glucose were the key indicators for enhancing the glycolysis/gluconeogenesis pathway in alfalfa; erythrose-4-phosphate was the key indicator for promoting the PPP pathway in alfalfa; and alpha-ketoglutarate was a key indicator for enhancing the TCA cycle in alfalfa. In amino acid biosynthesis, methionine and glutamate were the key indicators used to distinguish alfalfa drought resistance; in arginine and Pro metabolism, methionine, argininosuccinic acid, Spm and methionine sulfoxide were the key indicators used to distinguish alfalfa drought resistance; and in phenylpropanoid biosynthesis, cinnamic acid, *p*-coumaric acid and scopoline were the key indicators used to distinguish alfalfa drought resistance. In addition, TZ and SA were the key hormones used to distinguish drought resistance in alfalfa.

#### Mantel correlation heatmap between glycolysis/gluconeogenesis, the TCA cycle and other dams

**Fig. 11:**
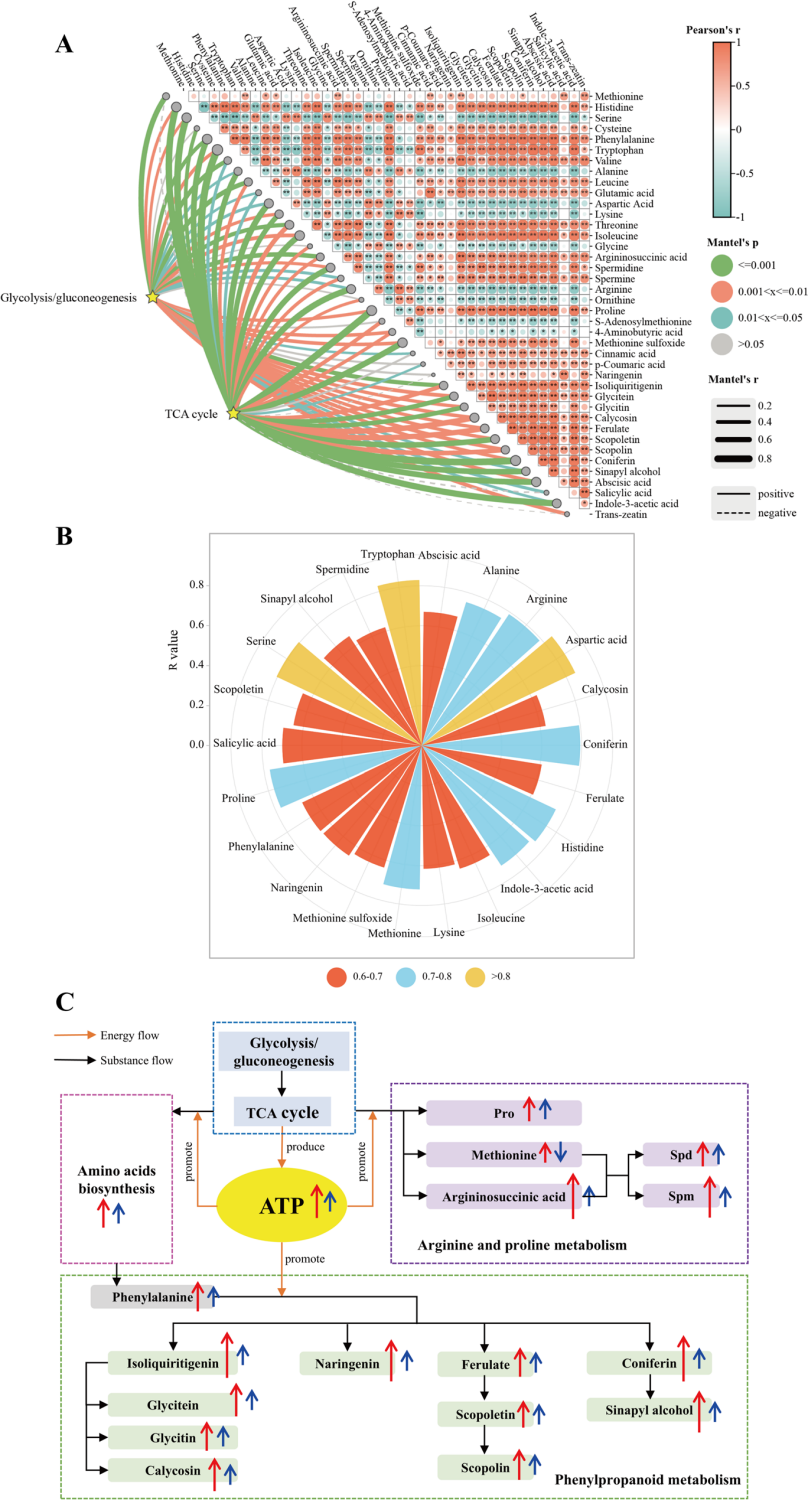
**A** Mantel correlation heatmap between glycolysis/gluconeogenesis, the TCA cycle and other DAMs. Correlations are significant at *p < 0.05 and **p < 0.01. **B** Metabolites with correlation coefficients *R* > 0.6 associated with glycolysis/gluconeogenesis and the TCA cycle. **C** Changes in metabolic pathways and metabolites under drought stress in two alfalfa varieties with different degrees of drought resistance. “↑” indicates an increase; “↓” indicates a decrease. The length of “↑” indicates the magnitude of change. “↑” in red represents the change in LZ; “↑” in blue represents the change in G3

We generated a mantel correlation heatmap between glycolysis/gluconeogenesis, the TCA cycle and other DAMs (Fig. 11A). The results revealed that glutamic acid was positively correlated with Pro (*p* < 0.01); arginine and s-adenosylmethionine were negatively correlated with Spd (*p* < 0.01) and Spm (*p* < 0.05); IAA was positively correlated with tryptophan (*p* < 0.01); and scopoletin was positively correlated with scopoline (*p* < 0.01). In addition, glycolysis/gluconeogenesis and the TCA cycle, which produce ATP, were positively correlated with other DAMs. These findings indicate that ATP from sugar metabolism is involved in amino acid biosynthesis, arginine and Pro metabolism, and phenylpropanoid biosynthesis. The metabolites positively correlated with glycolysis/gluconeogenesis and the TCA cycle with correlation coefficients R between 0.6 and 0.7 were phenylalanine, lysine, isoleucine, Spd, methionine sulfoxide, calycosin, ferulate, scopoletin, sinapyl alcohol, naringenin, salicylic acid and abscisic acid; R between 0.7 and 0.8 were histidine, alanine, arginine, proline, coniferin, methionine and indole-3-acetic acid; R greater than 0.8 were serine, tryptophan and aspartic acid (Fig. 11B). On the basis of the above results, we produced a diagram of metabolic pathway differences between alfalfa varieties with different drought resistance under drought stress (Fig. 11C). Compared with G3, LZ could maintain higher glycolysis/gluconeogenesis and the TCA cycle, and high glycolysis/gluconeogenesis and the TCA cycle provided more ATP and substrates for amino acid biosynthesis, arginine and proline metabolism, and phenylpropanoid biosynthesis. This allowed LZ to accumulate more drought resistance-related substances involved in amino acid biosynthesis, arginine and proline metabolism, and phenylpropanoid biosynthesis, such as amino acids, polyamines, and phenylpropanoids.

## Discussion

Drought stress is a dominant environmental factor limiting plant growth and development, crop yield and geographical distribution [20]. It has been shown that PEG-6000 can induce significant drought stress in plants but does not cause toxic effects or any physiological damage [21]. Thus, in our study, we used PEG-6000 to simulate drought stress.

### Phenotypic characteristics

Plant growth can be severely suppressed by drought stress [22]. Our results also revealed that the height of alfalfa plants decreased under drought stress. This occurred because the water content of plant tissues was reduced and cell extension was inhibited under drought stress. The plant root system plays a crucial role in water uptake. In most plants, root length increases under drought stress to promote water uptake [23]. In this study, drought stress resulted in increased root length in LZ; however, root length decreased in G3. This occurred because drought-resistant varieties receive more stress signals and can better adapt to drought stress by increasing their root length to obtain water from greater depths. A reduction in fresh and dry weight is a common deleterious effect of drought stress in plants [24]. Drought stress led to a decrease in the fresh and dry weights of single plants of both varieties of alfalfa, with a greater decrease in fresh and dry weights observed in G3. A previous study revealed that drought stress increased root length in drought-resistant rapeseed varieties but decreased root length in drought-sensitive rapeseed varieties. In addition, drought stress reduced the biomass of rapeseed, with drought-resistant varieties showing a lower decrease in biomass than drought-sensitive varieties [13]. This result was similar to our findings. The RWC is considered a measure of the water content of plant tissues, and plants with higher RWC are more resistant to drought stress [25]. Our results revealed that drought stress caused a decrease in RWC in both varieties of alfalfa, but LZ was able to maintain a greater RWC than G3. Similarly, drought-resistant sorghum [26] and apple rootstocks [27] presented higher RWC values under drought stress than did sensitive varieties.

### Glycolysis/gluconeogenesis, PPP and the TCA cycle

The glycolysis pathway converts glucose to pyruvate while producing a small amount of energy. In contrast, the gluconeogenesis process, which synthesizes glucose from pyruvate, shares some reversible reactions to the glycolysis pathway [28]. The glycolysis/gluconeogenesis pathway has been reported to respond to biotic and abiotic stresses mainly by affecting the ATP supply in plants [29]. ATP is the main form of energy in plants, providing energy for various metabolic activities, and cellular ATP deficiency often poses a threat to plant growth and development, crop quality, and stress resistance [30]. In this study, LZ was able to maintain higher glycolysis/gluconeogenesis under drought stress than G3. Pyruvate produced in the glycolysis/gluconeogenesis pathway is oxidized to acetyl-CoA, which then enters the TCA cycle. The TCA cycle can provide adequate ATP for plants to meet increased energy demands in response to drought stress and has been reported to be one of the core pathways for drought resistance in two soybean varieties [31]. In this study, LZ accumulated more metabolites related to the TCA cycle under drought stress than did G3. The above results indicated that maintaining higher glycolysis/gluconeogenesis and TCA cycle to provide adequate ATP was essential for improving drought resistance in alfalfa. These results were consistent with previous reports of enhanced glycolysis/gluconeogenesis pathway and TCA cycle in drought-resistant garden asparagus under drought stress [14]. Furthermore, a proteomic study of alfalfa under drought stress also revealed that drought-resistant alfalfa presented a relatively high abundance of proteins related to the glycolysis/gluconeogenesis pathway and the TCA cycle [32]. This result was further supported by our results at the metabolic level.

The PPP is an important pathway in plant sugar metabolism, and its major physiological function is to generate reduced NADPH and pentose phosphate to involve in nucleotides and amino acids synthesis [33]. Our results revealed that the PPP was significantly enriched under drought stress in both varieties. Compared with G3, LZ accumulated more metabolites involved in the PPP pathway, such as ribulose-5-phosphate, ribose-5-phosphate and erythrose-4-phosphate, under drought stress. Similar studies showed that the PPP in drought-resistant rice varieties [34] and heat-resistant chickpea varieties [35] was significantly enriched under stress conditions. In addition, NADPH can maintain GSH in a reduced state to prevent peroxidation of membrane lipids.

### Amino acid biosynthesis

Plants subjected to stress alter the levels of a series of free amino acids in their cells to better adapt to the environment [36]. Amino acids can act as important osmoregulators and ROS scavengers, maintaining the stability of the cell membrane structure, delaying protein degradation, and compensating for the lack of energy supply, thus improving the resistance of plants to stresses [13]. Amino acid biosynthesis is crucial for plant survival under drought stress [37]. A previous study revealed that amino acid accumulation played a key role in the regulation of drought resistance in apple rootstocks [27]. In our study, the drought-resistant variety LZ had the ability to accumulate more amino acids and prevent amino acid degradation under drought stress. This may be because LZ exhibited strong sugar metabolism, which provided a carbon skeleton and ATP for amino acid synthesis [38, 39]. The Mantel correlation analysis also supported this view (Fig. 11). In addition, our results showed that methionine accumulation was particularly important for enhancing drought resistance in alfalfa. A previous study revealed that high levels of Met were one of the signatures of drought resistance in broccoli [40]. This finding was consistent with our findings. Met may be a precursor to glucosinolates and other molecules (such as polyamines) involved in the stress response.

### Arginine and pro metabolism

Arginine and Pro metabolism are associated with nitrogen metabolism in plants and are essential for nucleic acid and protein synthesis as well as polyamine and glutathione biosynthesis [41]. The glutamic acid pathway is the main pathway for Pro production [42]. In the present study, drought stress caused LZ to accumulate more glutamic acid than G3, which led to a concomitant accumulation of more Pro in LZ. Correlation analysis also revealed that glutamic acid was positively correlated with Pro (*p* < 0.01). The contents of arginine and S-adenosylmethionine decreased in both LZ and G3 under drought stress, which was attributed to the synthesis of polyamines. This finding was also confirmed by the negative correlations of arginine and S-adenosylmethionine with Spd (*p* < 0.01) and Spm (*p* < 0.05). In addition, the arginine and S-adenosylmethionine contents of LZ decreased less than those of G3 did, probably due to the high accumulation of the precursors argininosuccinic acid and methionine in LZ. Spd and Spm are polyamines that favour plant resistance to drought stress [18]. Our results showed that drought stress induced LZ to accumulate more Spd and Spm. The finding that drought-resistant barley [43] and tomato [44] accumulate more Spd and Spm under drought stress was consistent with our results. The RDA results revealed that Spm played a more important role in enhancing drought resistance in alfalfa than did Spd. This occurred because Spm carries more nitrogen, which favours enhanced plant drought resistance [45].

GABA induces a variety of stress-responsive transcription factors that are involved in the expression of drought-responsive genes for signal transduction, osmoregulation and antioxidant [46]. The GABA level significantly decreased in G3 under drought stress, whereas LZ was able to maintain a stable GABA content. Similarly, drought-resistant rapeseed exhibited a relatively high GABA content [47]. Methionine can be oxidized by ROS to methionine sulfoxide (MetSO). MetSO causes changes in peptide hydrophobicity, alterations in protein conformation, and even loss of protein bioactivity [48]. In this study, drought resulted in a significant increase in MetSO content in both varieties of alfalfa, with a greater increase observed in G3. This may be because LZ exhibited higher methionine sulfoxide reductase activity, which reduced MetSO to methionine [49]. This speculation requires further investigation. Previous studies have shown that ATP facilitates the conversion of Met to the polyamine precursor S-adenosylmethionine [50], the conversion of glutamic acid to Pro [51], and the transport of polyamines [52]. This suggested that the higher arginine and Pro metabolism in LZ was partly due to the ability of LZ to produce more ATP than that produced by G3. This finding was further supported by the results of the mantel correlation analysis.

### Phenylpropanoid metabolism

The phenylpropanoid metabolic pathway begins with phenylalanine produced by the shikimate pathway, which first forms cinnamic acid and *p*-coumaric acid, followed by the production of *p*-coumaryl CoA, a common substrate of phenylpropanoids [53]. In this study, drought stress induced LZ to accumulate more phenylalanine and cinnamic acid than did G3, which provided more substrates for phenylpropanoids production. In addition, the results revealed that the *p*-coumaric acid content of G3 increased significantly under drought stress, but that of LZ did not change significantly. This may be the result of the high conversion of *p*-coumaric acid to phenylpropanoids in LZ.

Phenylpropanoids can be classified into five groups: flavonoids, phenolic acids, monolignols, stilbenes, and coumarins [54]. Studies have shown that flavonoids can mitigate damage caused by abiotic stress by scavenging ROS [55–57]. Naringenin, isoliquiritigenin, glycitein, glycitin, and calycosin are flavonoids, and our results revealed that drought stress induced the accumulation of these flavonoids in alfalfa, with a greater increase observed in LZ. Similarly, flavonoid levels in the hairy roots of alfalfa overexpressing *MsC3H29* were elevated under drought stress, increasing drought resistance [58].

Ferulate is a phenolic acid produced by the phenylpropanoid metabolic pathway. Ferulate has been reported to be one of the most powerful photoprotectants, providing protection for photosynthesis during drought stress [59]. Our results revealed that drought stress induced LZ to accumulate more ferulate compared with G3, which may be an important reason why LZ could maintain higher photosynthesis under drought stress than G3.

Scopoletin and its glucosylated form scopolin are coumarins that have antimicrobial and antioxidant activities and play essential roles in disease resistance [60], but few reports have linked them to abiotic stresses [61]. In this study, drought stress significantly increased the accumulation of scopolin in alfalfa, with greater accumulation observed in LZ. This may be due to the high accumulation of scopoletin in LZ. The significant positive correlation between scopolin and scopoletin also supports this hypothesis. The RDA results revealed that scopolin was the key coumarin involved in enhancing drought resistance in alfalfa Exogenous addition of scopolin may be considered as a strategy to enhance the drought resistance of alfalfa in practical production.

Lignin is a biopolymer of monolignols. The accumulation of lignin in alfalfa under drought stress has been reported to increase its drought resistance [62]. Sinapyl alcohol and coniferin are relevant metabolites for the synthesis of lignin. In this study, drought stress resulted in significant accumulation of coniferin and sinapyl alcohol in both varieties of alfalfa, with LZ accumulating more of these substances than G3. Similar studies showed that under drought stress, coniferin accumulated only in drought-resistant Siberian wildrye varieties [63]; waterlogging-resistant cotton varieties accumulated more sinapyl alcohol than waterlogging-sensitive cotton varieties to increase flooding resistance [64].

It has been reported that ATP can increase the accumulation of phenylpropanoids by increasing the activity of key enzymes in phenylpropanoid metabolism [65]. Mantel correlation analysis also revealed that phenylpropanoids were positively correlated with ATP-producing glycolysis/gluconeogenesis and the TCA cycle. Therefore, the maintenance of increased glycolysis/gluconeogenesis and TCA cycle activity and thus increased ATP production in LZ under drought stress was an important reason for the increased accumulation of phenylpropanoids.

### Plant hormones

Plant hormones act coordinately to regulate complex signalling pathways that ensure the optimal function of plant cell activity [66]. ABA and SA are key hormones involved in the plant response to drought, and they are significantly accumulated and initiate a variety of plant responses under drought stress, including stomatal closure and altered expression of stress resistance genes, which increase plant drought resistance [67, 68]. In this study, drought significantly increased the accumulation of ABA and SA in LZ compared to G3. Drought-resistant wheat [69] and salt-resistant soybean [70] could accumulate more ABA and SA under drought and salt stress, respectively. These findings were similar to our results. ABA has been demonstrated to regulate root architecture under drought stress and lengthen plant roots to allow them to reach water at greater depths [71]. The relatively high levels of ABA in LZ under drought stress may be an important reason for its relatively high root length. IAA has been shown to increase drought resistance in plants by regulating hormone levels, gene expression, antioxidant defence, and root structure [72]. Our results revealed that drought induced an increase in the IAA content in alfalfa. This may be because drought stress can upregulate IAA gene expression in alfalfa [73]. We noted that the accumulation of IAA was almost similar in the two varieties under drought stress. This may be due to the fact that tryptophan, the synthesized precursor of IAA, rose about the same amount in both varieties. Additionally, the significant positive correlation between IAA and tryptophan (*p* < 0.01) supported this speculation. TZ can increase photosynthesis, antioxidant enzymes activity, and osmoregulatory substances content in alfalfa, thereby reducing drought stress damage to plant cells [74]. Similarly, our results revealed that drought stress significantly increased the TZ content only in the drought-resistant variety LZ, suggesting that TZ is an important hormone involved in the resistance of alfalfa to drought stress. RDA revealed that TZ and SA were key hormones for enhancing drought resistance in alfalfa. It has been shown that SA, IAA and ABA are important phytohormones for drought stress resistance in alfalfa roots [16]. Our results showed that ABA, SA, IAA and TZ were plant hormones involved in the resistance of alfalfa leaves to drought stress, with SA and TZ playing more important roles. These results further lay a theoretical foundation for understanding the involvement of plant hormones in the resistance of alfalfa to drought stress.

## Conclusion

**Fig. 12:**
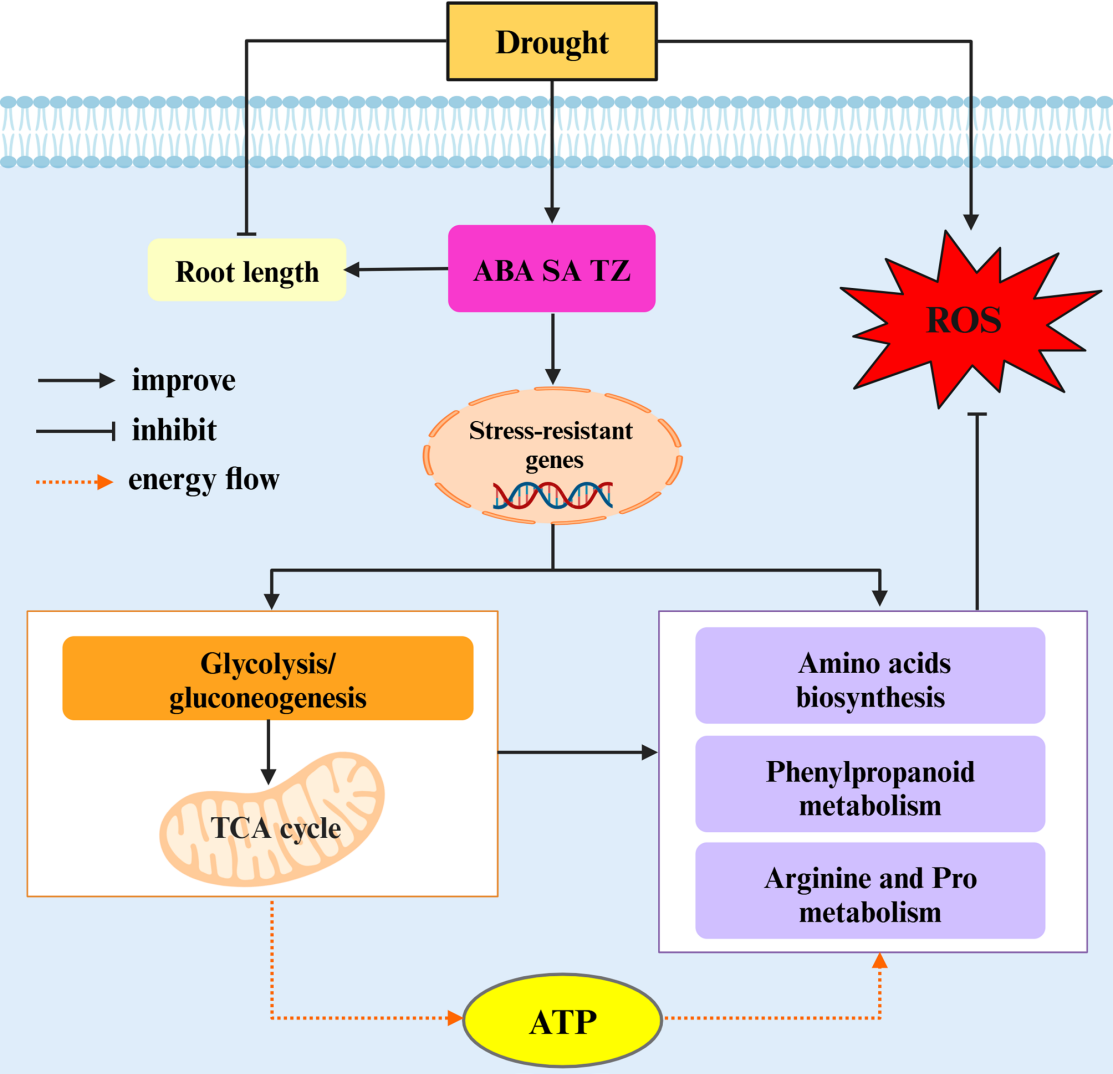
A schematic model of the drought resistance mechanism in alfalfa at the physiological and metabolic levels

In this study, we explored the mechanism of drought resistance in alfalfa by comparing the phenotypic, metabolic pathway, and metabolite differences between two different drought-resistant alfalfa varieties in response to drought stress. On the basis of the literature and our data, we proposed a schematic model for the drought resistance mechanism in alfalfa at the phenotypic and metabolic levels (Fig. 12). Drought stress could induce the accumulation of ABA, SA and TZ in drought-tolerant alfalfa leaves. ABA might promote root elongation in alfalfa under drought stress. ABA, SA and TZ might induce the expression of resistance genes, which resulted in the accumulation of amino acids, polyamines (Spd and Spm), flavonoids (naringenin, isoliquiritigenin, glycitein, glycitin and calycosin), phenolic acids (ferulate), coumarins (scopolin and scopoletin), and lignin synthesis precursors (sinapyl alcohol and coniferin), all of which enhance alfalfa drought resistance by effectively scavenging excess ROS. In addition, resistance genes might enhance glycolysis/gluconeogenesis and the TCA cycle, providing more substrates for the synthesis of drought-resistant metabolites related to amino acid biosynthesis, arginine and Pro metabolism and phenylpropanoid metabolism as well as providing sufficient ATP for various metabolic processes in alfalfa. These results revealed the mechanism of drought resistance in alfalfa at the phenotypic and metabolic levels, providing theoretical support for the breeding of drought-resistant alfalfa. However, how ABA, SA and TZ activate the expression of resistance genes and which resistance genes are activated to affect the accumulation of drought resistance metabolites need to be studied in depth. Our future work will focus on exploring the molecular mechanism of the metabolite response to drought stress through combined transcriptomic, genomics and targeted metabolomic analyses.

## Supplementary Information

Supplementary Material 1.

## Data Availability

Data is provided within the manuscript or supplementary information files.
